# LGD-4033 and a Case of Drug-Induced Liver Injury: Exploring the Clinical Implications of Off-Label Selective Androgen Receptor Modulator Use in Healthy Adults

**DOI:** 10.7759/cureus.69601

**Published:** 2024-09-17

**Authors:** Harrison Labban, Brittany Kwait, Awais Paracha, Mohammed Islam, Dolly O Kim

**Affiliations:** 1 Internal Medicine, Northwell Health, Queens, USA

**Keywords:** drug-induced acute liver failure, drug-induced liver injury (dili), sarm, severe acute liver toxicity, supplement-related liver injury

## Abstract

Selective androgen receptor modulators (SARMs), designed to treat conditions such as muscle wasting and osteoporosis, are widely used among healthy adults seeking muscle hypertrophy and enhanced athletic performance, despite a lack of Food and Drug Administration (FDA) approval. This trend may be driven by the misconception that SARMs are safer alternatives to anabolic steroids. However, SARMs such as LGD-4033 (Ligandrol) are associated with significant adverse effects, including hepatotoxicity, cardiovascular complications, endocrine disturbances, and psychiatric symptoms. This report examines the clinical implications of off-label SARM use, focusing on a case of drug-induced liver injury (DILI) in a 52-year-old male. The patient presented with pruritic jaundice, significant weight loss, and elevated liver enzymes following three months of high-dose LGD-4033 use. A diagnostic workup ruled out other potential causes of liver injury, implicating SARM use as the likely etiology. This case underscores the necessity for heightened clinical vigilance, early diagnosis, and prompt intervention to mitigate serious health outcomes associated with SARM misuse.

## Introduction

Selective androgen receptor modulators (SARMs) are a class of therapeutic compounds with similar anabolic properties to anabolic steroids but with reduced androgenic properties [1]. Despite their intended use in treating conditions such as muscle wasting and osteoporosis, there has been a concerning rise in the off-label use of SARMs, particularly among young men in the United States [2,3]. This demographic is often targeted with SARMs marketed to expedite bodybuilding, muscle gain, and fat loss, improve athletic performance, and achieve a hypermasculine physique [4]. However, the misuse of SARMs poses significant health risks, many of which are not yet fully understood [5].

Off-label SARM use is proliferating at an alarming rate, largely fueled by the misperception that SARMs are safer alternatives to anabolic steroids [4]. SARMs such as Ostarine, Ligandrol/LGD-4032, and RAD-140, while promising in clinical trials for specific conditions, have not been approved by the Food and Drug Administration (FDA) for general use due to their potential for serious side effects [6]. These include hepatotoxicity, cardiovascular issues, hormonal imbalances, and a range of psychiatric symptoms. The unregulated market for SARMs poses a particular risk, as the use of such products may be adulterated or mislabeled, leading to unpredictable and dangerous health outcomes [5,7].

A wide spectrum of pathologies can arise from the inappropriate use of SARMs and other over-the-counter (OTC) supplements marketed to increase muscle mass, burn fat, or promote endurance [8,9]. Hepatotoxicity is a well-documented adverse effect. This can manifest as elevated liver enzymes, jaundice, and in severe cases, acute liver failure [10]. Drug-induced liver injury (DILI) can be characterized by hepatocellular injury, cholestatic injury, or a mixed picture [5]. In patients with abnormal liver chemistry where DILI is suspected, the R factor, calculated as the ratio of serum alanine transaminase (ALT) to alkaline phosphatase (ALP), aids in differentiating the underlying pattern of injury [11]. Cardiovascular complications are also significant, with reports of myocardial infarction, hypertension, and hyperlipidemia associated with SARM use [12]. Furthermore, endocrine disturbances such as testosterone suppression can lead to long-term reproductive issues [13,14]. The psychological effects, including mood swings, aggression, and depression, add another layer of complexity to the clinical picture [7].

Diagnosing SARM-related adverse effects presents unique challenges. Many users may not disclose their SARM use due to stigma or legal concerns, leading to potential underreporting and misdiagnosis [15]. Symptoms of SARM-induced pathologies can mimic other common conditions, further complicating the diagnostic process. For instance, liver injury may be initially attributed to viral hepatitis or alcohol use, delaying appropriate intervention [5]. Moreover, metabolic dysfunction-associated steatotic liver disease (MASLD), previously known as non-alcoholic fatty liver disease (NAFLD), could also be overlooked in SARM users who present with abnormal liver chemistry [11]. Cardiovascular symptoms might be misdiagnosed as idiopathic or attributed to primary cardiac disease without considering the role of SARMs [5]. Timely intervention is critical, as prolonged exposure to SARMs can exacerbate the severity of adverse effects and complicate recovery.

The clinical implications of managing SARM-induced pathologies are profound. Physicians must maintain a high index of suspicion and consider off-label SARM use in differential diagnoses, particularly in patients presenting with unexplained hepatic, cardiovascular, or endocrine abnormalities. Early identification and cessation of SARM use are paramount in mitigating adverse effects. Follow-up care should be comprehensive, not only addressing the immediate health concerns but also monitoring for long-term sequelae such as chronic liver disease, persistent endocrine dysfunction, and potential future exposures with continued supplement use.

Here, we present the case of a 52-year-old male patient, previously in good health, who presented with pruritic jaundice and a rapid, unexplained weight loss of 15 pounds over the span of one week. Subsequent investigations confirmed the diagnosis of drug-induced liver injury most likely resulting from recent off-label use of SARMs. This case underscores the critical need for awareness and education regarding the risks associated with over-the-counter SARM use and highlights the importance of timely diagnosis and intervention in mitigating serious health outcomes.

## Case presentation

Clinical summary

A 52-year-old male with no reported past medical history presented to the emergency department with a one-week history of jaundice and pruritus. The patient reported an unintentional weight loss of 10-15 pounds over the past two weeks, accompanied by dark urine and yellow stools. He also experienced heartburn but denied any lethargy, abdominal pain, nausea, vomiting, chest pain, or shortness of breath. The patient's history was negative for alcohol abuse (consumption limited to 1-2 drinks per week), acetaminophen use, consumption of raw or undercooked foods, and recent travel. He had no family history of Gilbert's disease.

Notably, the patient reported having taken supplements containing LGD-4033, a selective SARM marketed for bodybuilding, at higher-than-recommended doses over the past three months, stopping two weeks prior due to running out of the product. He also admitted to taking these supplements for longer than the recommended duration but did not notice any side effects during usage. Additionally, he endorsed the use of pre-workout supplements and whey protein powders but denied the use of any other supplements.

The patient's vital signs revealed a blood pressure of 164/94 mmHg but were otherwise normal. On physical examination, the patient appeared jaundiced with scleral icterus but had normal mental status and abdominal examinations. He was well-nourished and of lean muscular build. Laboratory tests indicated significantly elevated bilirubin levels (direct bilirubin: 10 mg/dL, total bilirubin: 17 mg/dL), elevated liver enzymes (aspartate aminotransferase (AST): 107 U/L, alanine aminotransferase (ALT): 207 U/L), and elevated alkaline phosphatase (196 U/L) (Table 1). Urinalysis was positive for bilirubin. A computed tomography (CT) scan of the abdomen and pelvis revealed an incidental hepatic hemangioma but was otherwise unremarkable.

**Table 1:** Laboratory value trends AST: aspartate aminotransferase, ALT: alanine aminotransferase

Laboratory test	Value on admission	Value upon discharge (6 days later)	Value 3 months after discharge	Reference range
Bilirubin, total	17 mg/dL	12.2 mg/dL	1 mg/dL	0.2-1.2 mg/dL
Bilirubin, direct	10 mg/dL	8.2 mg/dL	0.3 mg/dL	0.0-0.3 mg/dL
Urine bilirubin	Positive	Small (positive)	Negative	Negative
AST	107 U/L	74 U/L	42 U/L	10-40 U/L
ALT	207 U/L	131 U/L	66 U/L	10-45 U/L
Alkaline phosphatase	196 U/L	146 U/L	103 U/L	40-120 U/L

These findings, particularly the elevated bilirubin and liver enzyme levels, suggested significant liver dysfunction, potentially cholestasis or hepatocellular injury. Given the patient's history of SARM use, a drug-induced cause ranked highly on our differential diagnosis.

Urinalysis confirmed the presence of conjugated hyperbilirubinemia, as indicated by the positive bilirubin test, supporting hepatic/biliary tract dysfunction. Imaging studies, including a CT scan of the abdomen and pelvis, revealed an incidental hepatic hemangioma but no other significant abnormalities that could explain the liver dysfunction.

To exclude viral hepatitis as a cause, a comprehensive viral hepatitis panel was performed, which returned negative for hepatitis A, B, C, D, and E. This result ruled out viral hepatitis. Autoimmune testing revealed a positive anti-smooth muscle antibody (ASMA), which suggested a possible autoimmune component. However, the absence of other autoimmune markers, such as a normal antinuclear antibody (ANA) and liver kidney microsomal (LKM) antibody, along with the clinical context, made autoimmune hepatitis less likely. The patient was also confirmed not to have recently taken any of the drugs implicated in drug-induced autoimmune-like hepatitis (DI-ALH).

Hemolysis was ruled out as a cause of jaundice through normal hemolysis laboratory results. Thyroid dysfunction was excluded with normal thyroid-stimulating hormone (TSH) levels. Further testing for additional viral infections, including cytomegalovirus (CMV) and Epstein-Barr virus (EBV), returned negative, excluding these infections as potential causes of liver dysfunction.

Iron studies, along with vitamin B12 and folate levels, were normal, ruling out iron overload and deficiencies that could contribute to liver disease. Coagulation studies indicated normal results, suggesting that despite liver dysfunction, the synthetic function of the liver was preserved.

Treatment

In the ED, the patient received 1 liter of normal saline intravenously, and the N-acetylcysteine (NAC) protocol was initiated as a prophylactic measure against potential acetaminophen toxicity, although the patient denied acetaminophen use. For pruritus management, the patient was started on cholestyramine and ursodiol to support liver function. The patient's reported gastroesophageal reflux (GERD) symptoms were treated with a proton pump inhibitor (PPI) leading to noticeable clinical improvement. Systemic corticosteroids were not administered, and their use in the management of DILI remains controversial [16], as discussed below.

Outcome and follow-up

The patient was discharged with instructions to follow up with hepatology for ongoing management of the mixed hyperbilirubinemia, likely induced by supplement use, and to monitor the incidental hepatic hemangioma. He was advised to seek immediate medical attention if he experienced worsening jaundice, dark urine, or yellowing of the eyes.

Liver function tests were down-trending by the time of discharge (bilirubin: 12.2 mg/dL, AST: 74 U/L, ALT: 131 U/L). Follow-up with serial serum bilirubin, AST, and ALT levels is crucial in this case to monitor the patient's ongoing liver function. Although not all patients with a diagnosis of DILI require hepatology follow-up after acute management, referral to a transplant center is necessary if there is evidence of acute liver failure. A significant sign of poor prognosis and potential need for future transplant is jaundice in the setting of ALT levels greater than three times the upper limit of normal. Since the patient in our case met this criterion, a hepatology follow-up was arranged.

He has no history of prior esophagogastroduodenoscopy (EGD) or red flag symptoms and was encouraged to follow up with outpatient GI if his reflux symptoms recur or worsen.

## Discussion

This case of a 52-year-old male presenting with significant hepatic dysfunction related to exposure to OTC workout supplements underscores the growing public health concerns of selective androgen receptor modulator (SARM) misuse. Despite their initial promise in clinical settings for conditions such as muscle wasting and osteoporosis, SARMs have become popular off-label among individuals seeking rapid muscle gain and fat loss [4]. This trend is especially pronounced among young men in the United States, driven by the perception that SARMs offer a safer alternative to anabolic steroids [4]. However, as evidenced by this case and supported by emerging literature, this belief is largely unfounded and potentially dangerous.

SARMs such as Ostarine, Ligandrol (LGD-4033), and RAD-140 have not received FDA approval for general use, primarily due to their potential for severe side effects [7]. The belief in their safety has been propagated by non-regulated markets where products are often mislabeled or adulterated. Studies have highlighted the risks associated with these compounds, including hepatotoxicity, cardiovascular issues, hormonal imbalances, and psychiatric symptoms [5,6,12-14]. The hepatotoxic effects observed in our patient, characterized by elevated liver enzymes and significant jaundice, as well as weight loss secondary to poor appetite, align with existing reports of liver damage from SARMs [9]. Additional symptoms of DILI can include malaise, low-grade fever, nausea and vomiting, and right upper quadrant pain, although our patient did not endorse these complaints [10].

Diagnosing SARM-induced liver injury presents unique challenges. Many users may not disclose their use due to stigma or legal concerns, leading to potential underreporting and misdiagnosis [4]. This patient's case was complicated by his initial reluctance to attribute his symptoms to SARM use. Symptoms such as jaundice and pruritus are nonspecific and can mimic other conditions such as viral hepatitis or autoimmune liver disease, complicating the diagnostic process [5,6]. DILI is primarily a diagnosis of exclusion, necessitating a robust differential and thorough workup. Comprehensive testing ruled out other causes, ultimately pointing toward SARM-induced hepatotoxicity.

Early identification and cessation of SARM use are critical in mitigating adverse effects [15]. The patient's treatment included supportive measures such as intravenous fluids and N-acetylcysteine (NAC), along with cholestyramine and ursodiol to manage pruritus and support liver function. The mainstay of treatment for DILI is the withdrawal of the offending drug [15], but at least one study [17] has demonstrated improved transplant-free survival with intravenous NAC given to non-acetaminophen toxicity patients experiencing early acute liver failure, notably, a more severe presentation in our patient, in which case NAC was removed once acetaminophen toxicity was eliminated from our differential. This approach highlights the need for clinicians to maintain a high index of suspicion for SARM use and broad differential in patients presenting with unexplained hepatic, cardiovascular, or endocrine abnormalities.

The role of systemic corticosteroids in the management of DILI remains controversial [16]. Glucocorticoids may be considered for patients with hypersensitivity reactions. Additionally, steroids are often administered to individuals exhibiting worsening cholestasis even after cessation of the offending drug or if histological findings suggest autoimmune hepatitis [15,18]. Hence, steroids may be administered when it is difficult to distinguish between autoimmune hepatitis and DILI or when a DILI event presents with prominent autoimmune hepatitis features [17,18]. No such features were present in this patient's case, making the use of systemic corticosteroids unnecessary.

The patient was advised to follow up with hepatology for ongoing management, reflecting the importance of monitoring for long-term sequelae such as chronic liver disease and persistent endocrine dysfunction. This case also underscores the critical need for increased awareness and education regarding the risks of over-the-counter SARM use. Public health initiatives should focus on the dissemination of accurate information about the potential dangers of these compounds and the importance of using medically supervised alternatives for muscle gain and performance enhancement.

This case illustrates the significant health risks associated with the misuse of SARMs and the challenges in diagnosing and managing their adverse effects. It emphasizes the need for heightened clinical awareness and public health education to mitigate the impact of SARM misuse on individual and public health.

## Conclusions

The off-label use of SARMs, such as LGD-4033, poses significant health risks, challenging the perception of these compounds as safer alternatives to anabolic steroids. The case presented here underscores the potential severity of SARM-induced liver injury and the broad spectrum of adverse effects linked to SARM misuse. Clinicians must maintain a high index of suspicion for SARM use in patients presenting with unexplained hepatic, cardiovascular, or endocrine abnormalities. Early identification and cessation of SARM use are crucial in mitigating adverse effects. Comprehensive follow-up care is essential to monitor for long-term sequelae and prevent future exposures. Public health initiatives should focus on educating the public about the dangers of unsupervised SARM use and promoting safer, medically supervised alternatives for muscle gain and performance enhancement. Enhanced clinical awareness and targeted public health education are vital in addressing the growing concerns associated with SARM misuse.
